# Antepartum acute Stanford type A aortic dissection: a case report and literature review

**DOI:** 10.1186/s13019-022-01817-7

**Published:** 2022-04-12

**Authors:** Shibo Song, Lin Lu, Lihua Li, Hua Peng, Xijie Wu

**Affiliations:** Department of Cardiac Surgery, Xiamen Cardiovascular Hospital of Xiamen University, School of Medicine, Xiamen University, Jinshan Road 2999, Huli District, Xiamen, China

**Keywords:** Pregnant woman, Aortic dissection, Antepartum/prepartum

## Abstract

**Background:**

Aortic dissection in pregnancy is a life-threatening event that is associated with high maternal and foetal mortality. Most cases occur during the third trimester of pregnancy, Herein, we describe a case of a pregnant woman with acute type A aortic dissection at 28 weeks of gestation.

**Case presentation:**

A previously healthy, 24-year-old gravida 2 para 1 woman was brought to the emergency department during at the 28 weeks of gestation and diagnosed with acute type A aortic dissection. Cesarean section was performed with the cardiac surgical team on standby for cardiopulmonary bypass and the patient delivered a baby weighing 1000 g. After the operation, we performed the Beatall procedure and total arch replacement with FET using the deep hypothermic circulatory arrest technique. Both the mother and child survived and recovered well. A review of the literature on antepartum acute aortic dissection during pregnancy is also presented.

**Conclusion:**

Women should have a comprehensive, systematic physical examination before getting pregnant. Women at high risks of aortic dissection must undergo multidisciplinary evaluation and be counseled before pregnancy, once they become pregnant, their consistent aortic root diameter should be consistently monitored, and their blood pressure strictly controlled.

## Introduction

Fetation potentially increases the risk of vascular disease, which is attributed to pregnancy hormonal and maternal hemodynamic changes and rein-angiotensin-aldosterone System [1]. According to the IRAD study, 1% of women with available data were diagnosed with pregnancy-related aortic dissection, and type A aortic dissection accounted for 45% of the cases [2]. Acute aortic dissection is a rare but life-threatening to both the mater and fetus, accounting for 19.8% of pregnancy-associated acute arterial dissection cases [3]. The incidence rate of aortic dissection or rupture was four times higher in the pregnant state than in the non-pregnant state [4].

We report a case of antepartum acute type A aortic dissection in a 25-year-old patient at 28 weeks of gestation who was successfully rescued and followed up at our cardiac surgery department. Meanwhile, a review of the literature of cases on prepartum acute type A aortic dissection was also done, aimed at describing the condition’s risk factors, timing, clinical characteristics, the maternal and foetal outcomes, intervention strategies, and how to acquire a good outcomes.

## Presentation of case

A previously healthy, 24-year-old gravida 2 para 1 woman was brought to the emergency department during at the 28 weeks of gestation with sudden-onset rigorous chest pain radiating to the back. She had delivered uneventfully 2 years earlier but had subclinical hypothyroidism and gestational diabetes. She was 168 cm tall and weighed 80 kg. On initial presentation, her blood pressure was 148/73 mmHg, heart rate was 74 beats/min, respiratory rate was 23 breaths/min, and temperature was 36.5 °C. Her D-dimer level was elevated (4.32 mg/L), and her cardiac enzymes (including troponin T) were normal. Cardiovascular examination revealed a diastolic murmurs in the aortic valve area. The foetal heart rate was 155 beats/min without signs of foetal distress, and the woman did not experience uterine contractions. She displayed almost all the classic clinical manifestations of Marfan syndrome (MFS) (tall, thin appearance, arachnodactyly, funnel chest, ectopia lentis), but had no family history of aortic dissection. Considering the potential harm of the intravenous contrast agent used in the aortic artery angiography, only echocardiography was performed. Transthoracic echocardiography showed acute Stanford type A aortic dissection from the ascending aorta to the iliac artery with an ascending aortic aneurysm and a dilated aortic root up to 46 mm (Fig. 3).

The patient was on medications and she was hemodynamically stable, with a normal blood pressure and heart rate with the help of medication. After consultation among the cardiac surgery, obstetrics, neonatology, and anesthesiology teams, cesarean section was performed followed by aortic repair. The abdomen was closed before cardiopulmonary bypass (CPB), and hysterectomy was not performed because the bleeding was controlled. Meanwhile, the cardiac surgical team was on standby for CPB in the operating room in case of an aortic emergency during cesarean. A male baby was delivered with and resuscitated by endotracheal intubation, and admitted to the neonatal intensive care unit where surfactant was administered.

After the cesarean section, the cardiopulmonary bypass was built by cannulating from the right atrium and perfusing into the femoral artery and axillary artery. The Bentall procedure (mechanical valve replacement and coronary artery reimplantation) and total arch replacement with a tetrafurcate graft with stented elephant trunk implantation was performed [5]. The patient did not undergo genetic testing, so she was not diagnosed with any genetic disease.

The patient was admitted to the intensive care unit and she recovered uneventfully. On postoperative day 12, she was discharged from the hospital on oral anticoagulation with warfarin (her international normalized ration was 2–2.5).

On postoperative day 30, she was found to have her right lateral hemiplegia and logagnosia. A computerized tomography scan of the head revealed an acute infarction in the left cerebellar hemisphere (Fig. 2). The patient did not go any surgical intervention; she underwent rehabilitation training and was soon transferred to the ward and later discharged from the neurology department.

## Discussion

We briefly reviewed 37 cases in the literature to determine if there are similarities with our successful case and to offer a more comprehensive consultation for pregnant women who are at high risk of aortic dissection. It is widely known to all that pregnancy-specific changes increase the risk of aortic dissection in pregnant women. Physiological changes in pregnancy, such as an increased heart rate, stroke volume, cardiac output, ventricular dimensions, and fluctuations in oestrogen and progesterone levels, significantly exert hemodynamic stress on the aortic wall, and this peaks in the third trimester [1]. In our review, type A aortic dissection most frequently occurred in the third trimester (30 cases, 81.1%).

**Fig. 1:**
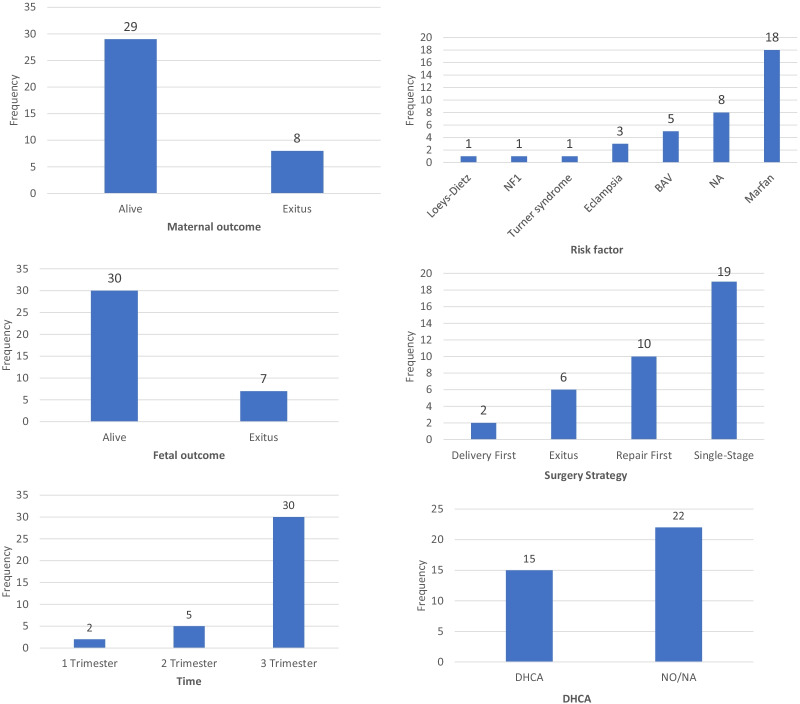
Histogram of the data on maternal outcomes, fetal outcomes, timing, risk factors, surgical strategy, and deep hypothermia circulatory arrest. The frequency indicates the number of cases from a total of 37 cases

**Fig. 2:**
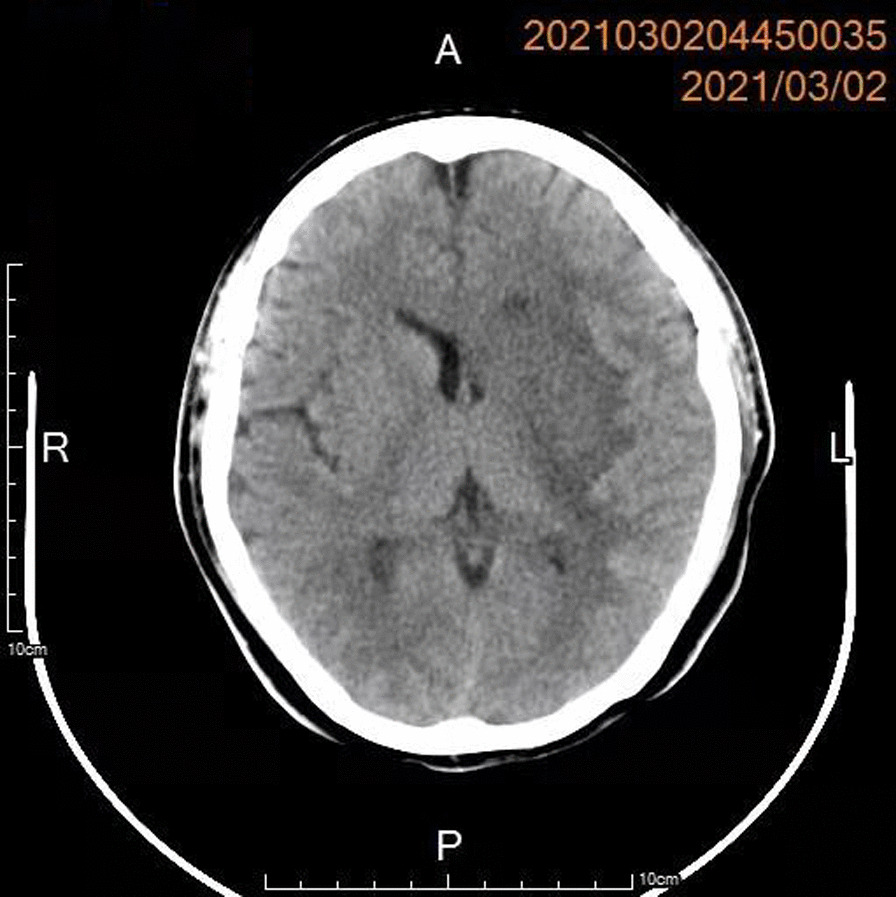
A cerebral infarction in the left basal ganglia one month later after cesarean section and aortic repair

The descriptive statistics of the data from the case reports as well as frequencies and percentages of the total cases are shown in Table 1. The previously published literature on prepartum acute type A aortic dissection, including symptoms at onset, surgical strategy and risk factors is presented in Table 2. A histogram of the data on maternal outcomes, foetal outcomes, timing, risk factors, surgical strategies and deep hypothermic circulatory arrest is shown in Fig. 1. Cerebral infarction in left basal ganglia one month later after cesarean section and aortic repair is shown in Fig. 2.

**Table 1:** Descriptive statistics of the data from the case reports as well as frequencies and percentages of the cases

Item	Mean/frequency	Percentage (%)
Cases	37	/
Age	32.1	
Timing		
1st Trimester	2	5.4
2nd Trimester	5	13.5
3nd Trimester	30	81.1
Risk factor		
Loeys-Dietz syndrome	1	2.7
NF1	1	2.7
Turner syndrome	1	2.7
Eclampsia	3	8.1
BAV	5	13.5
NA	8	21.6
Marfan syndrome	18	48.6
Presentation		
Epigastric/back/chest pain	29	78.4
Dyspnea	6	16.2
Shock	1	2.7
Hand weakness and dyspraxia	1	2.7
Pleural effusion	1	2.7
NA	5	13.5
Sudden death	4	10.8
Surgical strategy		
Single-stage delivery and repair	19	51.4
Repair first	10	27.0
Delivery first	2	5.4
Exitus	6	16.2
CPB manner		
DHCA	15	40.5
NA/NO	22	59.5
Maternal outcome		
Alive	29	78.4
Exitus	8	21.6
Foetal outcome		
Alive	30	81.1
Exitus	7	18.9

NF-1, neurofibromatosis type1; BAV, bicuspid aortic valve; NA, not available; DHCA, deep hypothermic circulatory arrest

**Table 2:** The previously published literature on prepartum acute type A aortic dissection: symptoms at onset, surgical strategy, and risk factors

Author (year)	Age	Gestational week	Chief compliant	Aortopathy	Risk factors	Surgical strategy	Maternal outcome	Fetal outcome
Lee [17]	35	37	Epigastric pain	DAA	NA	CS + AR	Survived	Survived
Wang [18]	33	28	Chest pain	DARS:52 mm	Marfan	CS + AR	Survived	Survived
Wang [18]	30	32	Chest pain and dyspnea	AS, AI, DARS:52 mm	BAVD	CS + AR	Survived	Survived
Murphy [19]	34	34	Chest pain and dyspnea	NA	Preeclampsia	CS + AR	NA	NA
Aziz [20]	30	28	Chest pain	BAV	BAV	CS + AR	Survived	Survived
Nonga [21]	29	29	Chest and back pain	DARS:60 mm	Marfan	CS + AR	Survived	Survived
Mohammad [22]	36	35	Chest pain, dyspnea	DARS:60 mm	NA	CS + AR	Survived	Survived
Crowley [23]	34	37	Chest pain, dyspnea	AI	NA	CS + AR	Survived	Survived
Yang [24]	31	33	Chest pain	AS, AI, DARS:50 mm	BAVD	CS + AR	Survived	Survived
Kim [25]	32	30	Chest and back pain	AI, DARS:52 mm	Marfan	CS + AR	Survived	Survived
Kim [25]	31	29	Chest pain	AI, DAR	Marfan	CS + AR	Survived	Survived
Seeburger [26]	29	17	NA	DAR + AAA	Marfan	AR	Survived	Survived
Gurbuz [27]	41	34	Epigastric pain and limb swelling	NA	Eclampsia	NA	Died	Survived
Kunishige [28]	32	16	Chest pian	DARS:65 mm	Loeys-Dietz syndrome	AR	Survived	Survived
Tateishi [12]	42	30	Chest pain and pleural effusion	Ruptured site, left pleural effusion	NF1	AR + CS	Survived	Survived
Yang [29]	35	33	Back pain	DAR	Marfan	CS	Survived	Survived
Yang [29]	33	12	Chest Pain	DAR	Marfan	Abortion first + AR	Died	Died
Pagni [30]	29	34	Chest pain	DARS:40 mm	Marfan	CS + AR	Survived	Survived
Nasiell [31]	30	36	Back pain	Pericardial effusion	NA	CS	Died	Survived
Nasiell [31]	40	38	Chest pain	NA	NA	CS + AR	Survived	Survived
Nasiell [31]	37	41	Shock	Degenerative disorde	NA	CS	Died	Survived
Sakaguchi [15]	32	33	NA	DARS: 35 mm	Marfan	CS + AR	Survived	Survived
Sakaguchi [15]	33	26	NA	DARS: 55 mm	Marfan	AR*	Died	Died
Sakaguchi [15]	28	30	NA	DARS: 85 mm	Marfan	AR + vaginal delivery	Survived	Survived
Sakaguchi [15]	34	34	NA	DARS: 60 mm	Marfan	CS + AR	Survived	Survived
Wakiyama [32]	36	21	NA	DARS: 60 mm	Marfan	AR	Survived	Died
Shaker [33]	34	7	Chest and back pain	NA	Marfan	AR	Survived	Survived
Vranes [34]	30	26	Chest pain	DAR	Marfan	AR*	Survived	Survived
Houston [35]	23	27	Chest pain and emesis	DAR	Marfan	AR + CS	Survived	Survived
Akhtar [36]	35	27	Chest pain	NA	NA	AR	Survived	Died
Ch’ng [37]	30	37	Cough and dyspnoea	NA	Marfan	CS + AR*	Survived	Survived
Ch’ng [37]	36	32	Chest pain	NA	NA	CS + AR*	Died	Survived
Ch’ng [37]	28	37	Left hand weakness and dyspraxia	NA	Marfan	CS + AR*	Survived	Survived
Ch’ng [37]	36	21	Epigastric pain	NA	Turner-syndrome BAV	Abortion first	Survived	Died
Ch’ng [37]	36	32	Pleuritic chest pain	NA	BAV	CS + AR*	Survived	Survived
Shetty [38]	21	8	Chest pain	NA	NA	NA	Died	Died
Ventura [39]	35	41	Chest and back pain	NA	NA	NA	Died	Died

DAA, dilated ascending aorta; NA, not available; CS, cesarean section; AR, aortic repair; DARS, dilated aortic root size; AS, aortic stenosis; AI, aortic insufficiency; BAVD, bicuspid aortic valve disease; AR*, aortic repair for rescue; CS + AR*, cesarean section (sternotomy standby at the same time) + aortic repair

### Risk factors

In the review,three pregnant women were diagnosed with Loeys-Dletz syndrome, neurofibromatosis type1, and Turner syndrome, respectively. Of the 18 women with MFS in the present series were with Marfan syndrome, 5 pregnant women had bicuspid aortic valves, and, 3 cases presented with eclampsia before dissection. It has been massively reported that connective tissue disorders such as MFS, LDS, NF-1 and Turner syndrome are strongly associated with acute aortic dissection. Of these, nearly half of the cases (18/37, 48.6%) were diagnosed with MFS, which was consistent with the previous reports [6].

MFS is a systemic connective tissue disorder that involves the eyes, bones and cardiovascular system. The diagnosis is based on the revised Ghent nosology and can be definitely made by gene testing. The current guidelines focus on increased aortic root diameter as an obvious risk factor for aortic dissection. The risk is estimated to be 1% when the diameter of the aortic root is less than 40 mm and could increase to 10% when the diameter is greater than 40 mm [7]. In our analysis, we found that the majority of the cases of aortic artery dissection were in pregnant women with MFS who had aortic root diameters > 40 mm and the US guidelines as well as Canadian and European guidelines suggest that women with MFS undergo prophylactic aortic repair before conception when aortic root diameter ≥ 40 mm and ≥ 45 mm, respectively. Furthermore, patients with MFS with who are growing fast are more likely to undergo aortic dissection during pregnancy. Interestingly, Katherine Smith [8] found a few numbers of pregnant women diagnosed with aortic dissection with aortic root diameter < 45 mm, and there must be underlying factors behind this phenomenon, thus further evaluation is needed. Vania Volach reported that successful pregnancy and delivery can be achieved in patients with MFS after root replacement [9]. However, Dominique Williams claimed that women with MFS who became pregnant following aortic root replacement were at high risk for distal aortic dissection, although the exact risk is difficult to quantify. Pregnant women with MFS should be counselled and their aortic root diameter should be followed regularly.

Loeys-Dietz syndrome, caused by mutations in TGFBR1 and TGFBR2, is characterized by vascular and skeletal abnormalities and arterial tortuosity, aneurysms and aortic dissection are the common presentations [10]. Braverman emphasize the high risk associated with pregnancy following root replacement in Loeys-Dietz, and patients should be counselled accordingly [11]. Our patient presented with a dilated aortic root measuring 65 mm.

Aortic stenosis, regurgitation, and dilatation of the aortic root are common features of a bicuspid aortic valve(BAV), which is founded in 1–2% of the population. In our review, 13.5% of the patients with acute type A aortic dissection had bicuspid aortic valves.

Neurofibromatosis type 1 is an autosomal dominant disorder that affects 1 in 3000 individuals.NF-1 may dominantly involve any tissue of the body, including connective tissue, nerve tissue, and vasculature. Two pathogenic mechanisms may account for the uncommon spontaneous aortic rupture in these cases: smooth muscle dysplasia and direct vascular invasion by neurofibromatous tissue, as well as ganglioneuromatous tissue invading the arterial wall. There was one case of spontaneous ascending aortic rupture in a pregnant woman with NF-1 in our review [12].

### Maternal and fetal outcome

The maternal and foetal outcome hospital mortality rates were 21.6% and 13.5%, respectively. Most of the dissections (81.7%) occurred in the third trimester: only nearly 20% occurred in the first and second trimester. In the IRAD study, the maternal hospital mortality rate was 3% [2]. Two pregnant women were died at 8 and 41 weeks of gestation before being admitted to the hospital.

### Presentational symptoms and diagnosis

Chest and back pain with/without radiation to other parts are the classic classical symptoms of aortic dissection. It can also manifest as several entities, including myocardial infarction, pulmonary embolism and limb weakness, or even hemianopia. A few paitents have been found to be in shock at presentation to the hospital and they had no chance for surgery. Thoraco-abdominal artery angiography is the “gold standard” of invasive aortography; however, exposure of the embryo to contrast agent is a concern. Iodinated contrast agents can suppress foetal thyroid function. Transthoracic echocardiography is a practical, non-invasive, bedside, and timely recommended diagnostic tool for unstable patients for whom there is a high degree of suspicion for aortic dissection.

### Surgical strategy

**Fig. 3:**
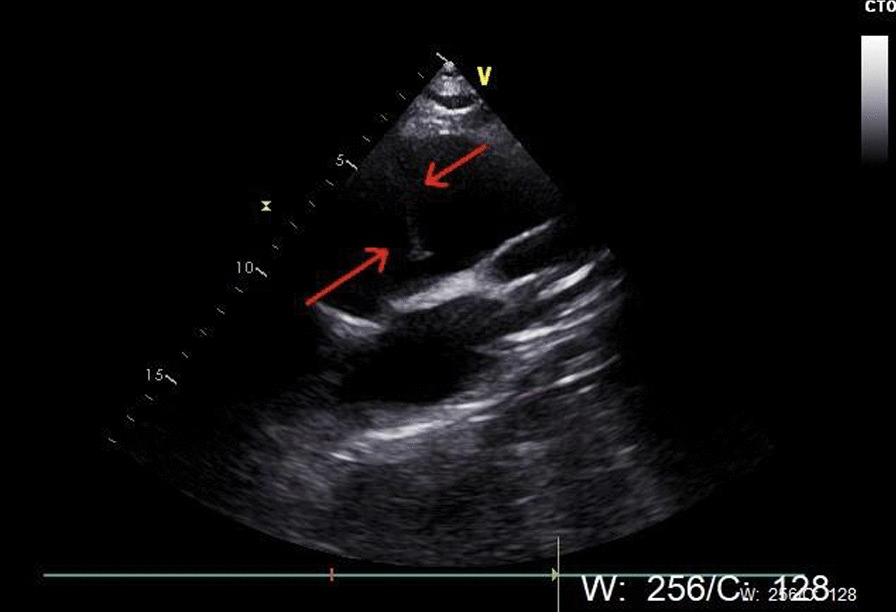
A flap floating in the aorta in the long axis of the aorta on transthoracic echocardiography

For acute type A aortic dissections, after 28 weeks of gestation weeks, delivery followed by surgical repair can achieve maternal and foetal survival; before 28 weeks of gestation, maternal survival should be prioritised given the high risk of foetal death; between 28 and 32 weeks, physicians should consider the risks to both mother and fetus [13]. A 21-year clinical experience in patients with MFS demonstrated that before 28 gestational weeks, aortic repair should be performed first, followed by maternal and foetal monitoring; after 28 gestational weeks, single-stage delivery and aortic repair is preferred [14]. In our analysis, all cases in their third trimester underwent a single-stage procedure, mostly with delivery first. Additionally, cardiovascular operation using deep hypothermia with total circulatory arrest for aortic repair may be associated with a higher risk of foetal mortality [15]. Some CPB parameters are adjusted to improve foetal outcome: for example, high flow rates and a target MAP > 70 mmHg are recommended for placental perfusion [16]. Three pregnant women in the first two trimesters underwent repair first, and successfully gave birth to healthy infants. The other cases were as follows: one pregnant woman chose abortion first, one patient died of multiple organ dysfunction and low cardiac output after repair, and the remaining two cases had no chance for repair (Fig. 3).

## Conclusion

Women should not be pregnant without a comprehensive and systematic physical examination. Women at high risks of aortic dissection must undergo multidisciplinary evaluation and counselling before pregnancy, and once they become pregnant, their aortic root diameter should be consistently monitored and their blood pressure strictly controlled.

## Data Availability

All data generated or analysed during this study are included in this published article.
